# Клинические рекомендации «Преждевременное половое развитие»

**DOI:** 10.14341/probl12821

**Published:** 2021-09-24

**Authors:** В. А. Петеркова, И. Л. Алимова, Е. Б. Башнина, О. Б. Безлепкина, Н. В. Болотова, Н. А. Зубкова, Н. Ю. Калинченко, М. А. Карева, А. В. Кияев, А. А. Колодкина, И. Б. Кострова, Н. В. Маказан, О. А. Малиевский, Е. М. Орлова, Е. Е. Петряйкина, Л. Н. Самсонова, Т. Е. Таранушенко

**Affiliations:** Национальный медицинский исследовательский центр эндокринологии; Смоленский государственный медицинский университет; Северо-Западный государственный медицинский университет имени И.И. Мечникова; Национальный медицинский исследовательский центр эндокринологии; Саратовский государственный медицинский университет им. В.И. Разумовского; Национальный медицинский исследовательский центр эндокринологии; Национальный медицинский исследовательский центр эндокринологии; Национальный медицинский исследовательский центр эндокринологии; Уральский государственный медицинский университет; Национальный медицинский исследовательский центр эндокринологии; Детская республиканская клиническая больница им. Н.М. Кураева; Национальный медицинский исследовательский центр эндокринологии; Башкирский государственный медицинский университет; Национальный медицинский исследовательский центр эндокринологии; Республиканская детская клиническая больница; Российская медицинская академия непрерывного профессионального образования; Красноярский государственный медицинский университет имени профессора В.Ф. Войно-Ясеневского

**Keywords:** клинические рекомендации, преждевременно половое развитие, дети

## Abstract

Преждевременное половое развитие является актуальной проблемой детской эндокринологии, характеризующейся клинической и патогенетической гетерогенностью. Появление вторичных половых признаков ранее 8 лет у девочек и 9 лет у мальчиков требует своевременной диагностики и назначения патогенетически обоснованного лечения с целью достижения целевых показателей конечного роста и предотвращения социальной депривации. Разработанные клинические рекомендации являются основным рабочим инструментом практикующего врача. В них кратко и структурированно изложены основные сведения об эпидемиологии и современной классификации преждевременного полового развития, методах его диагностики и лечения, базирующихся на принципах доказательной медицины.

## Abstract (en)

The precocious puberty is an urgent problem of pediatric endocrinology characterized by clinical and pathogenetic heterogeneity. The appearance of secondary sex characteristics before the age of 8 years in girls and 9 years in boys requires timely diagnosis and the appointment of pathogenetically justified treatment in order to achieve the target indicators of final growth and prevent social deprivation. The developed clinical guidelines are the main working tool of the practitioner. They briefly and structurally present the main information about the epidemiology and modern classification of рrecocious puberty, methods of its diagnosis and treatment based on the principles of evidence-based medicine.

СПИСОК СОКРАЩЕНИЙ

ТЕРМИНЫ И ОПРЕДЕЛЕНИЯ

## 1. КРАТКАЯ ИНФОРМАЦИЯ ПО ЗАБОЛЕВАНИЮ ИЛИ СОСТОЯНИЮ.

1.1. Определение заболевания или состояния.

Преждевременное половое развитие (ППР) — это появление вторичных половых признаков у девочек до 8 лет и у мальчиков до 9 лет [1–4].

1.2. Этиология и патогенез заболевания или состояния.

Выделяют гонадотропин-зависимую (центральную, истинную), гонадотропин-независимую (периферическую) и парциальную формы ППР.

Гонадотропин-зависимое ППР обусловлено преждевременной активацией центрального звена гипоталамо-гипофизарно-гонадной системы — повышение секреции половых стероидов гонадами при данной форме является следствием стимуляции половых желез гонадотропными гормонами гипофиза. Причиной преждевременного полового развития центрального генеза являются опухоли хиазмально-селлярной области, органическое поражение ЦНС (арахноидальные кисты, травмы, гидроцефалия), генетические нарушения (активирующие мутации гена-рецептора кисс-пептина GPR54 или гена кисс-пептина KISS1 и инактивирующие мутации гена MKRN3, переданного от отца) [1][5]. Центральное ППР с манифестацией в 6–7 лет характерно для синдрома Вильямса, синдрома однородительской дисомии 14 хромосомы материнского происхождения (синдрома Темпла), отмечено при синдроме Сильвера–Рассела [6–9]. Также преждевременная активация гонадолиберин-гонадотропов может возникнуть в ответ на избыток половых стероидов, возникающий при периферических формах ППР [10].

В большинстве случаев причина центрального ППР остается неизвестной, такие случаи называются идиопатическим гонадотропин-зависимым ППР.

Гонадотропин-независимое (периферическое) ППР обусловлено секрецией половых стероидов гонадами или опухолями гонад или надпочечников, избыточной секрецией андрогенов надпочечниками вследствие нарушений стероидогенеза [11].

Активация гонад без стимулирующего воздействия гонадотропинов и в отсутствие опухолей может быть следствием генетических нарушений (тестотоксикоз и синдром МакКьюна-Олбрайта-Брайцева (синдром МОБ)), воздействия на половые железы ХГЧ при герминогенных опухолях или ТТГ при декомпенсированном первичном гипотиреозе.

Тестотоксикоз — заболевание с аутосомно-доминантным типом наследования, возникает вследствие активирующих мутаций в гене LHCGR, кодирующем рецептор ЛГ. Постоянная активация рецептора приводит к стимуляции клеток Лейдига и синтезу тестостерона в яичках, несмотря на низкий уровень ЛГ [12, 13].

Синдром МОБ обусловлен активирующими соматическими мутациями гена GNAS, кодирующего стимулирующую альфа-субъединицу G-белка (Gas). Эта субъединица играет важную роль в передаче сигнала от множества пептидных гормонов в клетках-мишенях, в частности от ЛГ и ФСГ. Активация Gas приводит к повышению цАМФ в клетках гонад и продукции половых стероидов в отсутствие стимуляции гонадотропинами [14][15]. Клоны клеток с мутантными аллелями распределяются в гонадах неравномерно. У девочек они могут локализоваться в одном или двух яичниках, обусловливая периодическое появление эстрогенпродуцирующих кист с последующим их регрессом. У мальчиков также может возникать периферическое ППР, но чаще наблюдается изолированный макроорхидизм, что отмечается при преимущественном наличии мутаций в клетках Сертоли с меньшим содержанием или отсутствием таковых в клетках Лейдига [16–18].

Кроме синдрома МОБ, вариантом автономной гиперфункции половых желез у девочек является преходящее периферическое ППР вследствие функциональных кист яичников, причины появления которых остаются неясными [19][20].

ХГЧ-секретирующие герминогенные опухоли — объемные образования, возникающие вследствие нарушения дифференцировки плюрипотентных зародышевых клеток, возникающие в половых железах или вне гонад по срединной линии тела по пути миграции примордиальных зародышевых клеток [21]. Вырабатываемый опухолью ХГЧ, сходный по структуре с ЛГ, активирует рецепторы к ЛГ в клетках Лейдига в яичках и приводит к повышению секреции тестостерона. При этом, как правило, не происходит значимого увеличения в объеме яичек, так как клетки Сертоли, обеспечивающие сперматогенез, активируются под воздействием ФСГ [22]. ХГЧ-секретирующие герминогенные опухоли вызывают преждевременное половое развитие у мальчиков, тогда как для девочек с этим заболеванием ППР не характерно. Это объясняется тем, что биосинтез эстрогенов в яичниках требует воздействия как ЛГ, так и ФСГ. Тем не менее в литературе есть описание двух случаев периферического ППР у девочек с ХГЧ-секретирующими герминогенными опухолями [23][24].

Декомпенсированный первичный гипотиреоз также может быть причиной гонадотропин-независимого ППР. Наиболее вероятным механизмом развития ППР в этом случае считается стимуляция значительно повышенным ТТГ рецепторов к ФСГ в гонадах, что становится возможным благодаря наличию одинаковых альфа-субъединиц в структуре ТТГ и ФСГ [25][26]. Впервые ППР, обусловленное тяжелым первичным гипотиреозом, было описано в 1960 г. Ван Виком и Громбахом и теперь известно как синдром Ван Вика–Громбаха [27].

Опухоли надпочечников чаще секретируют андрогены, вызывая изосексуальное ППР у мальчиков и гетеросексуальное ППР у девочек, редко могут секретировать эстрогены, что приводит к развитию гетеросексуального преждевременного полового развития у мальчиков и изосексуального преждевременного полового развития у девочек. Среди стероид-секретирующих опухолей яичек чаще встречаются лейдигомы; опухоли яичников могут секретировать как эстрогены, так и андрогены.

Избыточная секреция андрогенов надпочечниками вследствие нарушений стероидогенеза при различных вариантах врожденной дисфункции коры надпочечников приводит к периферическому ППР по типу изосексуального у мальчиков и гетеросексуального у девочек.

Полные формы ППР характеризуются системным воздействием половых гормонов на организм ребенка, что проявляется прогрессирующим развитием вторичных половых признаков и ускорением костного созревания с увеличением скорости роста.

Парциальные формы ППР представляют собой преждевременное изолированное увеличение молочных желез у девочек (изолированное преждевременное телархе) и преждевременное изолированное развитие полового оволосения (изолированное преждевременное адренархе) [1][28].

Изолированное увеличение молочных желез у девочек рассматривается как следствие транзиторной активации центрального звена гипоталамо-гипофизарно-гонадной оси с преобладающим избытком ФСГ [29].

Изолированное преждевременное адренархе возникает вследствие преждевременного созревания сетчатой зоны коры надпочечников с повышением уровня дегидроэпиандростерон сульфата (ДГЭА-С), обладающего относительно слабыми андрогенными свойствами. Патогенез преждевременного адренархе остается неясным, на данный момент лишь установлено, что девочки с этим состоянием находятся в группе риске по развитию синдрома поликистозных яичников [30].

1.3. Эпидемиология заболевания или состояния (группы заболеваний или состояний).

Распространенность ППР зависит от нозологического варианта, пола и возраста. Гонадотропин-зависимые формы ППР встречаются у девочек значительно чаще, чем у мальчиков. По данным мировой литературы, распространенность центральных форм ППР у девочек до 2 лет составляет 0,5 случая на 10 000 детского населения, от 2 до 4 лет — 0,05:10 000, от 5 до 9 лет — 8 :10 000. Среди мальчиков ППР встречается реже вне зависимости от возраста — 0,05: 10 000 [31].

Гонадотропин-независимое ППР встречается гораздо реже, распространенность зависит от формы заболевания.

1.4. Особенности кодирования заболевания или состояния по Международной статистической классификации болезней и проблем, связанных со здоровьем.

E30.1 Преждевременное половое созревание.

E22.8 Преждевременное половое развитие центрального происхождения.

E28.1 Избыток андрогенов.

E29.0 Гиперфункция яичек.

E31.1 Полигландулярная гиперфункция.

1.5. Классификация заболевания или состояния.

В основу классификации синдрома ППР положен патогенетический принцип, учитывающий первичную локализацию патологического процесса в системе гипоталамус–гипофиз–гонады–надпочечники.

1. Гонадотропин-зависимое ППР.

## 2. Гонадотропин-независимое преждевременное половое развитие.

2.1. Обусловленное секрецией половых стероидов гонадами.

У девочек:

У мальчиков:

2.2. Обусловленное секрецией половых стероидов опухолями гонад или надпочечников.

2.3. Обусловленное избыточной секрецией андрогенов надпочечниками вследствие нарушений стероидогенеза (ВДКН) — всегда изосексуальное у мальчиков и гетеросексуальное у девочек.

## 3. Изолированные формы преждевременного полового развития.

3.1. Изолированное преждевременное телархе.

**Table:** Таблица 1. Классификация преждевременного полового развития

Уровень поражения	Нозологический вариант	Соответствие полу
Гонадотропин-зависимое (центральное) преждевременное половое развитие	Гипоталамическая гамартома	Всегда изосексуальное
Опухоли хиазмально-селлярной области (астроцитома, глиома и т.д.)
Врожденные аномалии ЦНС (гидроцефалия, арахноидальные кисты, супраселлярные кисты, гранулематозные заболевания)
Приобретенные травмы ЦНС (хирургическое вмешательство, облучение, травма, воспаление)
Генетически обусловленное (мутации в генах KISS1, GPR54, MKRN3)
Ассоциированное с синдромальной патологией (синдром Вильямса, синдром однородительской дисомии 14 хромосомы материнского происхождения, синдром Сильвера–Рассела)
Предварительный избыток половых стероидов, возникающий при периферических формах ППР
Гонадотропин-независимое преждевременное половое развитие, обусловленное автономной секрецией половых стероидов гонадами	Мутации в гене GNAS — синдром МОБ: эстрогенпродуцирующие кисты яичников у девочек/автономная гиперсекреция тестостерона в яичках у мальчиков/макроорхидизм без автономной гиперсекреции тестостерона у мальчиков	Всегда изосексуальное
Фолликулярные кисты яичников
Мутации в гене LHCGR — семейный тестотоксикоз
ХГЧ-секретирующие герминомы
Первичный гипотиреоз (синдром Ван Вика-Громбаха)
Гонадотропин-независимое преждевременное половое развитие, обусловленное опухолями гонад и надпочечников	Эстроген-продуцирующие опухоли яичников	Изосексуальное у девочек
Андроген-продуцирующие опухоли яичников	Гетеросексуальное у девочек
Лейдигомы	Изосексуальное у мальчиков
Эстроген-продуцирующие опухоли надпочечников	Гетеросексуальное у мальчиков и изосексуальное у девочек
Гонадотропин-независимое преждевременное половое развитие, обусловленное нарушениями стероидогенеза	Формы врожденной дисфункции коры надпочечников (дефицит 21-гидроксилазы)	Гетеросексуальные у девочек и изосексуальные у мальчиков
Неполные (изолированные) формы преждевременного полового развития	Преждевременное изолированные телархе	Изосексуальное у девочек
Преждевременное изолированные адренархе	Изосексуальное у мальчиков и гетеросексуальное у девочек (надпочечникового генеза)

3.2. Изолированное преждевременное адренархе.

Истинное гонадотропин-зависимое преждевременное половое развитие всегда бывает полным, т.е. у девочек после увеличения молочных желез и ускорения темпов роста появится половое оволосение и наступит менархе, а у мальчиков наряду с увеличением гениталий и ускорением роста будет происходить увеличение объема яичек.

При гонадотропин-независимом половом развитии у мальчиков происходит андрогенизация, однако нет стимулирующего воздействия ФСГ на гонады, вследствие чего не отмечается роста объема яичек; у девочек возможны нециклические менструалоподобные кровотечения, обусловленные резкими колебаниями уровня эстрогенов при кистах яичников или при синдроме МОБ.

Особенности клинической картины при различных нозологических формах ППР

Гипоталамическая гамартома (ГГ) — наиболее часто выявляемое образование центральной нервной системы (ЦНС) у детей с истинным ППР до 3 лет жизни вне зависимости от пола. ГГ не является опухолью, а представляет собой врожденную эктопию гипоталамической ткани. ГГ в 70% случаев вызывает ППР [32]. Помимо ППР, гамартомы гипоталамической локализации могут сопровождаться неврологическими и поведенческими аномалиями. Типичным проявлением интрагипоталамического (сесильного) типа гамартом является приступы насильственного смеха (gelastic seizures). Часто отмечаются эмоциональная лабильность, агрессивность, снижение памяти, возможно снижение интеллекта [33][34].

Глиомы и астроцитомы как причина истинного ППР встречаются значительно реже. Большинство глиом, сопровождающихся клинической картиной ППР, локализуются в области хиазмы и дна 3-го желудочка или распространяются вдоль зрительного тракта. Подавляющее число глиом обладает низкой пролиферативной активностью и представляют собой доброкачественные пилоидные астроцитомы, обладающие тенденцией к медленному росту. Анапластические астроцитомы (злокачественный вариант глиомы) встречается преимущественно у взрослых. На МР-снимках глиомы представляют собой однородную массу, сходную по плотности с веществом мозга. Оптические глиомы небольших размеров могут манифестировать только симптомами ППР, большие размеры опухоли хиазмы и дна 3-го желудочка могут сопровождаться явлениями несахарного диабета, СТГ-дефицита, сужением полей зрения, снижением остроты зрения и общемозговыми симптомами, связанными с повышением внутричерепного давления. Достаточно часто симптомы ППР, или ускоренного пубертата, развиваются сразу после оперативного удаления или облучения глиом хиазмально-селлярной локализации [35].

Синдром МакКьюна–Олбрайта–Брайцева — мультисистемное заболевание, компонентами которого являются пятна цвета «кофе с молоком», фиброзная дисплазия скелета, гиперфункция эндокринных желез (гонад, соматотрофов гипофиза, фетальной коры надпочечников, щитовидной железы), а также ряд патологий других органов (тахикардия, холестатический гепатит, гастроинтестинальные полипы, внутрипротоковая папиллярно-муцинозная опухоль поджелудочной железы) [17][36]. Фиброзная дисплазия проявляется деформациями верхних конечностей, деформациями нижних конечностей по типу пастушьего посоха, деформациями черепа, патологическими переломами костей, сопровождается гиперфосфатурией с возможностью развития гипофосфатемического рахита [37]. Характерной особенностью периферического ППР при синдроме МОБ у девочек является волнообразное течение с непредсказуемой частотой появления признаков ППР в виде увеличения молочных желез с/или без кровянистых выделений из половых путей [16][17][38]. У мальчиков при синдроме МОБ чаще наблюдается макроорхидизм без других признаков ППР и в отсутствие повышения тестостерона [16–18].

Активирующие мутации гена рецептора к ЛГ приводят к периферическому ППР только у мальчиков, и такой вариант заболевания называется тестотоксикоз. Его отличительной чертой является высокий уровень тестостерона при подавленных уровнях гонадотропинов и при размере яичек, не соответствующем повышенным значениям тестостерона (не более 6–8 мл), поскольку основной объем яичек составляют структуры, рост которых стимулируется ФСГ. При данной нозологии отмечается ранняя манифестация (на первом году жизни) и быстрая прогрессия признаков полового развития [12][13].

ХГЧ-секретирующие герминогенные опухоли. В детском возрасте встречаются ХГЧ-секретирующие гепатобластомы, реже медиастинальные тератомы и тератобластомы, ретроперитонеальные карциномы, хориокарциномы и герминомы гонад. Для краниальных ХГЧ-секретирующих опухолей наиболее типичная локализация — пинеальная область, реже супраселлярная цистерна. До 30% герминативно-клеточных опухолей имеют злокачественный характер. Краниальные ХГЧ-секретирующие опухоли характеризуются многообразной неврологической симптоматикой. При супраселлярной локализации в неврологической симптоматике преобладают симптомы повышения внутричерепного давления и зрительные нарушения, связанные с поражением оптической хиазмы. Возможны эндокринные нарушения: с высокой частотой встречается несахарный диабет, реже — СТГ-дефицит. При опухолях, локализующихся в пинеальной области, ведущей является неврологическая симптоматика, обусловленная сдавлением тенториума: нистагм, парез взора вверх, анизокория, а также выраженные симптомы внутричерепной гипертензии [39].

Синдром Ван Вика–Громбаха характеризует ППР на фоне декомпенсированного первичного гипотиреоза. У девочек отмечаются кровянистые выделения из половых путей, реже — увеличение молочных желез и галакторея, у мальчиков — увеличение в объеме яичек. Костный возраст, как правило, отстает. После назначения заместительной терапии левотироксином натрия симптомы ППР регрессируют [25–27].

Однородительская дисомия 14 хромосомы материнского происхождения, делеции 14 хромосомы отцовского происхождения и нарушения метилирования дифференциально метилируемых регионов 14 хромосомы приводят к развитию синдрома Темпла (по имени автора, впервые описавшего это заболевание в 1991 г.), включающего в себя: дефицит веса, пре- и постнатальную задержку роста, гипотонию, акромикрию кистей и стоп, умеренные проявления лицевого дисморфизма, легкую степень задержки умственного развития, преждевременное половое развитие с ранним менархе, которое наступает в возрасте 8–10 лет, сопровождающееся опережением костного возраста в отсутствие значимого ускорения роста [7][8].

Синдром Вильямса (СВ) — это мультисистемное заболевание, обусловленное делециями длинного плеча 7 хромосомы. При СВ отмечается ряд фенотипических черт (характерное «лицо эльфа», пухлые губы и хриплый голос), поведенческих особенностей (чрезмерно дружелюбное поведение, умственная отсталость) и патологий различных органов и систем (надклапанный аортальный стеноз, стеноз периферических легочных артерий, аномалии развития почек, конечный рост ниже среднего и др.). Из эндокринных нарушений при СВ отмечаются гиперкальциемия с манифестацией в первые годы жизни, гипотиреоз, нарушения углеводного обмена, центральное преждевременное половое развитие с манифестацией в возрасте 6–7 лет [6].

Преждевременное изолированное телархе характеризуется увеличением молочных желез до 3 стадии развития по Tanner, без ускорения костного возраста и прогрессии других вторичных половых признаков [29, 40]. Чаще всего возникает в течение первых двух лет жизни и в большинстве случаев регрессирует. После 2 лет также возможно возникновение изолированного телархе, однако в таких случаях повышен риск трансформации изолированного телархе в полную центральную форму ППР [29].

Преждевременное изолированное адренархе не является признаком истинного центрального ППР, так как процесс активации сетчатой зоны коры надпочечников, где образуются андрогены, не регулируется гонадотропными гормонами. Клиническими признаками преждевременного адренархе являются типичные проявления гиперандрогении в виде появления лобкового оволосения, запаха пота и угревой сыпи, также характерно ускорение костного возраста [29][30][41]. Преждевременное адренархе — это диагноз-исключение, который может быть установлен только после обследования на предмет других возможных причин гиперандрогении.

## 2. ДИАГНОСТИКА ЗАБОЛЕВАНИЯ ИЛИ СОСТОЯНИЯ.

Диагностика ППР происходит поэтапно. На первом этапе необходимо констатировать наличие преждевременного полового развития, выделить группу неполных форм, т.н. изолированное телархе и адренархе. На втором этапе у пациентов с подтвержденным преждевременным половым развитием необходимо установить нозологический вариант с целью определения тактики лечения.

2.1. Жалобы и анамнез.

- Сроки появления молочных желез у девочек: появление молочных желез на первом году жизни чаще встречается при изолированном телархе [40][42] УДД — 4, УУР — С.

- Скорость прогрессии вторичных половых признаков: для овариальных эстрогенпродуцирующих кист яичников характерно интермиттирующее течение полового развития [43–46]. УДД — 4, УУР — С.

- Скорость роста за предшествующее 3–6 мес: при преждевременном изолированном телархе и адренархе нет ускорения роста. Отсутствие ускорения роста наблюдается при ППР на фоне гипопитуитаризма при арахноидальных кистах, септо-оптической дисплазии, опухолях ЦНС, после облучения ЦНС и при пороках развития гипоталамо-селлярной области. [47–49]. УДД — 3, УУР — B.

- Наличие признаков частых компонентов синдрома МакКьюна-Олбрайта-Брайцева: фиброзная дисплазия и/или пятна цвета «кофе с молоком» с неровными «географическими» краями [16][17][50–52]. УДД — 4, УУР — С.

- Наличие неврологической симптоматики и симптомов несахарного диабета: преждевременное половое развитие может быть последствием органического поражения ЦНС [53][54]. УДД — 4, УУР — С.

- Наличие признаков синдромальной патологии (возможные синдром Вильямса, Сильвера-Рассела, Темпла): стигмы дисэмбриогенеза, отставание в развитии, низкорослость, поведенческие особенности, пороки развития сердца, гиперкальциемия, признаки мышечной гипотонии, низкорослость и т.д.

2.2. Физикальное обследование.

-антропометрию с целью оценки коэффициента стандартного отклонения по росту — превышение роста более 2SD относительного целевого роста на данный возраст является критерием ППР [5][28][47]. УДД — 5, УУР — С;

-подсчет скорости роста за предшествующие 6–12 мес с целью определения скорости роста — увеличение скорости роста более 2SD за предшествующий период свидетельствуют в пользу ППР [5][28][47]. УДД — 5, УУР — С.

Комментарии.

Дополнительно при осмотре можно выявить сопутствующие симптомы, характерные для отдельных нозологических форм:

- Наличие пятен цвета «кофе с молоком» с неровными географическими краями характерно для синдрома МОБ.

- Наличие округлых/овальных пятен цвета «кофе с молоком», веснушек в подмышечных областях, нейрофибром характерно для нейрофиброматоза 1 типа, при котором развитие ППР обусловлено глиомами зрительного нерва.

- Наличие деформаций костей конечностей по типу пастушьего посоха, деформаций и асимметрий черепа характерно для синдрома МОБ.

- Наличие неврологической симптоматики (нистагм, косоглазие, шаткость походки и т.п.) может свидетельствовать в пользу органического поражения ЦНС.

- Нерегулярные менструалоподобные реакции характерны для синдрома МОБ.

2.3. Лабораторные диагностические исследования.

Комментарии.

-Наибольшей информативностью в диагностике гонадотропин-зависимого ППР обладает уровень ЛГ, но только при использовании высокочувствительных методик (ICMAС чувствительностью 0,01 Ед/л или ECLIA с чувствительностью 0,1 Ед/л) и наличии в лаборатории допубертатных нормативов.

- Значение ЛГ выше 0,3 Ед/л при использовании вышеуказанных методик с высокой вероятностью свидетельствует о наличии гонадотропин-зависимого ППР [57–60].

- Значение ЛГ менее 0,3 Ед/л не позволяет исключить гонадотропин-зависимое ППР, так как до 50% здоровых девочек со второй стадией полового развития имеют допубертатный уровень ЛГ [60][62–64].

- Уровень ЛГ у здоровых девочек младше 2 лет может соответствовать пубертатным значениям в соответствии с характерным для этого возраста «мини-пубертатом», что следует иметь в виду при интерпретации результатов [65][66].

- У девочек в возрасте до 3 лет с пальпируемыми молочными молочными железами повышенный уровень ФСГ при допубертатных значениях ЛГ указывает на наличие неполной формы ППР — преждевременного телархе [67][68].

Комментарии. При клинической картине, соответствующей стадии полового развития по Tanner 3 и выше, в сочетании с пубертатным уровнем ЛГ, в проведении пробы с аналогом Гн-РГ нет необходимости.

Методика проведения пробы с гонадотропин-рилизинг-гормоном: определяется базальный уровень ЛГ и ФСГ крови, вводится аналог ГнРГ короткого действия, на фоне стимуляции которым определяется в динамике уровень ЛГ и ФСГ крови. Препараты ГнРГ, использующиеся для пробы, и временные точки забора крови представлены в таблице 2.

**Table:** Таблица 2. Варианты проведения пробы с препаратом гонадотропин-рилизинг-гормона

Препарат	Доза	Способ введения	Время забора крови	Определяемые гормоны
#Бусерелин**	300 мкг	Интраназально	0, 1 ч, 4 ч	ЛГ и ФСГ
#Трипторелин**	100 мкг	П/к	0, 1 ч, 4 ч

Критерии оценки пробы с гонадотропин-рилизинг-гормоном: повышение ЛГ более 6 Ед/л на фоне стимуляции свидетельствует в пользу гонадотропин-зависимого ППР [60][61][68–74].

В инструкции к применению #трипторелина** 0,1 мг и #бусерелина** 150 мкг/доза нет указания на применение в рамках диагностических тестов при обследовании по поводу нарушений полового развития. Однако данные препараты применяются с указанной выше целью вне зарегистрированных показаний на основании результатов эффективности и безопасности по международным данным и клиническим опытом в ФГБУ «НМИЦ эндокринологии» [75–80].

Ранее пороговым значением ЛГ на пробе с аналогом ГнРГ, подтверждающим центральное ППР, считалось значение 10 Ед/л, однако в соответствии с данными международных исследований и клиническим опытом в ФГБУ «НМИЦ эндокринологии», рекомендовано снизить пороговое значение до 6 Ед/л [60][61][68–74].

Отсутствие повышения ЛГ свидетельствует в пользу гонадотропин-независимого ППР.

Превышение уровня ФСГ над уровнем ЛГ свидетельствует в пользу изолированного телархе. Исследование уровня общего тестостерона крови у мальчиков, исследование уровня общего эстрадиола у девочек позволяет подтвердить диагноз ППР при выраженной прогрессии полового развития и не является информативным на ранних стадиях истинного преждевременного полового развития.

Значительное превышение уровня общего эстрадиола крови на фоне допубертатных значений гонадотропных гормонов является критерием эстроген-секретирующих кист и опухолей яичников [17][19][20][44–46, 81].

Значительное превышение уровня андрогенов крови на фоне допубертатных значений гонадотропных гормонов является критерием гиперфункции яичек, опухолей яичек или надпочечников [11][13].

Комментарии.

- При идиопатическом преждевременном адренархе уровень ДГЭА-С (ДГЭА-С менее вариабельный показатель по сравнению с ДГЭА) соответствует 2 стадии по Tanner (40–135 мкг/дл или 1,1–3,7 мкмоль/л), тогда как при андроген-продуцирующих опухолях надпочечников ДГЭА-С превышает 500 мкг/дл (15 мкмоль/л).

- Уровень 17ОНР выше 15 нмоль/л (5нг/мл) с высокой вероятностью свидетельствует о неклассической форме дефицита 21-гидроксилазы. В сомнительных случаях (уровень 17ОНР 6–15 нмоль/л) рекомендовано дообследование (изложены в протоколах по ВДКН).

2.4. Инструментальные диагностические исследования.

Комментарии.

- Определение костного возраста проводится на основании рентгенографии кистей (левой кисти для правшей) — опережение костного возраста более чем на 2 года от паспортного является критерием ППР [90][95][96][98][99].

- При определении костного возраста рекомендуется использовать атлас Greulich-Pale, или систему Tanner-Whitehouse, или метод Бухмана [90][92][93][96].

Комментарии.

- Обязательно проведение МРТ у всех мальчиков с гонадотропин-зависимым ППР и у девочек младше 6 лет.

- Девочкам с дебютом гонадотропин-зависимого ППР в промежутке от 6 до 8 лет МРТ проводится при наличии неврологической симптоматики и/или признаков гипопитуитаризма [3][102].

- Поскольку гонадотропин-зависимое ППР не связано с аденомами гипофиза, нет необходимости в контрастном усилении при проведении МРТ.

Комментарии:

- наличие эхо-признаков фолликула более 1 см в диаметре у девочки препубертатного возраста определяется как киста яичника;

- при проведении УЗИ органов малого таза в рамках диагностики ППР следует оценивать соответствие размеров матки и объема яичников возрасту. Объем яичников рассчитывается по формуле: длина (см) × ширина (см) × высота (см) × 0,5233. При центральном ППР у девочек отмечаются бÓльшие размеры матки и объем яичников (по сравнению с препубертатным контролем и девочками с изолированным телархе) [107];

- высокоспецифичным, но малочувствительным признаком гонадотропин-зависимого преждевременного полового развития является наличие эхо-признаков эндометрия [43];

- у девочек на стадии манифестации гонадотропин-зависимого ППР матка и яичники могут быть еще допубертатных размеров, в связи с чем при дифференциальной диагностике между гонадотропин-зависимым преждевременным половым развитием и изолированным телархе проведение УЗИ матки и яичников у девочек может использоваться только как вспомогательный метод [43][108][109].

2.5 Иные диагностические исследования

Комментарии. Пациентам с синдромом МОБ необходимо регулярное обследование с целью скрининга компонентов заболевания:

- УЗИ щитовидной железы для исключения узлов.

- Исследование уровня свободного тироксина (св.Т4) крови, исследование уровня тиреотропного гормона (ТТГ) в крови для исключения тиреоидной автономии.

- Исследование уровня соматотропного гормона в крови, исследование уровня пролактина крови, исследование уровня инсулиноподобного ростового фактора 1 в крови (ИФР-1) для исключения акромегалии и гиперпролактинемии

- Оценка уровня тубулярной реабсорбции фосфатов для исключения гипофосфатемического рахита.

- Исключение синдрома Кушинга у детей первых лет жизни (исследование уровня адренокортикотропного гормона в крови (АКТГ), исследование уровня общего кортизола крови, исследование уровня свободного кортизола в моче мочи, исследование уровня дегидроэпиандростерона сульфата в крови (ДГЭА-С).

- Проведение глюкозотолерантного теста с определением СТГ (по показаниям — при повышенном уровне СТГ и/или ИФР-1).

- МРТ головного мозга для исключения аденомы гипофиза (по показаниям — при отсутствии подавления СТГ на пробе или при гиперпролактинемии).

- Сцинтиграфия костей всего тела для уточнения распространенности фиброзной дисплазии (после 5–6 лет) и/или рентгенография в прямой проекции нижних конечностей для исключения очагов фиброзной дисплазии, угрожаемых патологическими переломами и/или требующих оперативного лечения.

- МСКТ головы (по показаниям — для уточнения очагов фиброзной дисплазии черепа).

- Офтальмоскопия (по показаниям — для исключения атрофии зрительных нервов при наличии очагов фиброзной дисплазии черепа).

## 3. ЛЕЧЕНИЕ, ВКЛЮЧАЯ МЕДИКАМЕНТОЗНУЮ И НЕМЕДИКАМЕНТОЗНУЮ ТЕРАПИИ, ДИЕТОТЕРАПИЮ, ОБЕЗБОЛИВАНИЕ, МЕДИЦИНСКИЕ ПОКАЗАНИЯ И ПРОТИВОПОКАЗАНИЯ К ПРИМЕНЕНИЮ МЕТОДОВ ЛЕЧЕНИЯ

3.1. Консервативное лечение.

Терапевтическая тактика определяется этиологическим вариантом ППР. Целью лечения ППР является торможение прогрессирования костного возраста и препятствие прогрессии полового развития, что позволит адаптировать пациентов с психологической и социальной точек зрения.

Комментарии.

- Возможно назначение пролонгированных аналогов ГнРГ после 8 лет у девочек и 9 лет у мальчиков при наличии приобретенного СТГ дефицита на фоне опухолей ЦНС в том случае, если есть возможность терапии препаратами гормона роста (H01AС: Соматропин Гормоны передней доли гипофиза и их аналоги) для улучшения ростового прогноза.

- Пациентов с гонадотропин-зависимой формой ППР следует включать в Федеральный регистр лиц, страдающих жизнеугрожающими и хроническими прогрессирующими редкими (орфанными) заболеваниями, приводящими к сокращению продолжительности жизни граждан или их инвалидности» [121].

- В группе девочек с гонадотропин-зависимым преждевременным половым развитием в возрасте от 6 до 8 лет достоверного улучшения ростового прогноза не получено. Вопрос о назначении терапии в данной группе пациентов решается индивидуально с учетом психологической составляющей [122].

- В России на сегодняшний день зарегистрированы для использования у детей с гонадотропин-зависимым преждевременным половым развитием два препарата из группы пролонгированных аналогов ГнРГ для ежемесячных инъекций: #трипторелин** 3,75 мг и #лейпрорелин** 3,75 мг. Оба препарата имеют схожую эффективность и безопасность. Препараты трипторелин** или лейпрорелин** назначаются в/м 1 раз в 28 дней в стартовой дозе 3,75 мг вне зависимости от веса ребенка [123–127].

- В России имеются пролонгированные аналоги ГнРГ более длительного действия — трипторелин**11,25 мг и #лейпрорелин** 11,25 мг, которые назначаются в/м с частотой 1 раз в 12 нед. Эти препараты используются в Европе и США в лечении детей с преждевременным половым развитием с доказанной эффективностью и безопасностью [3][120].

Комментарии. Оценка эффективности проводится не раньше, чем через 3 мес от начала лечения, затем не реже 1 раза в год. Оценка результата лечения проводится только по совокупности клинических и лабораторных показателей [3][129].

Протокол ведения пациентов, получающих терапию пролонгированными аналогами ГнРГ:

Критерии эффективности терапии.

1.Скорость роста (снижение до возрастной нормы).

2.Отсутствие прогрессии полового развития или регресс вторичных половых признаков.

3.Прогрессия костного возраста не более чем на 1 год за 1 год (проводится 1 раз в год, при необходимости через 6 мес).

4.Базальный уровень ЛГ и стероидных гормонов крови (общего тестостерона для мальчиков, общего эстрадиола для девочек) может служить критерием эффективности только в том случае, если до начала лечения имелся повышенный уровень этих гормонов; умеренно повышенный уровень ФСГ не является признаком отсутствия эффекта от терапии.

В сомнительных случаях через 6 месяцев от начала терапии (при неполном соответствии критериям эффективности терапии) проводится проба с аналогом ГнРГ (за 1 день до очередной инъекции аналога Гн-РГ), критерием эффективности лечения является отсутствие выброса ЛГ на пробе более 4 Ед/л [130–137]. Считается доказанным отсутствие негативного влияния длительной терапии пролонгированными аналогами ГнРГ на набор веса и снижение минеральной плотности костной ткани у детей с ППР [129].

В настоящее время не существует четких критериев, определяющих сроки отмены терапии, каждый случай рассматривается индивидуально, принимая во внимание следующие критерии [3]:

- Достижение пубертатного возраста (девочки 10–12 лет, мальчики 11–13 лет).

- Достижение костного возраста (девочки 12–13 лет, мальчики 14 лет).

- Снижение скорости роста менее 2 SD для данного костного возраста.

- Достижение роста, соответствующего костному возрасту (конечный прогнозируемый рост, близкий к целевому).

- Психологическая готовность ребенка и родителей.

Лечение гонадотропин-независимого ППР.

1.Детям с ППР вследствие врожденной дисфункции коры надпочечников рекомендуется проводить (обследование и лечение) в соответствии с клиническими рекомендациями по ВДКН [138]. УДД — 5, УУР — С.

2.Детям с гонадотропин-независимыми формами ППР вопрос о назначении лечения рекомендуется решать в каждом случае индивидуально в специализированных центрах [11][139]. УДД — 5, УУР — С.

Комментарии. В настоящее время используются ингибиторы ароматазы 3 поколения — #летрозол, #анастрозол**; антиэстрогены — #тамоксифен** или #фулвестрант**; антиандрогены — #бикалутамид**; при этом не существует единых общепринятых схем и доз терапии гонадотропин-независимых форм преждевременного полового развития данными препаратами, но по данным мировой практики при лечении гонадотропин-независимого преждевременного полового развития #летрозол используют в дозе 2,5 мг ежедневно перорально, #анастрозол** 1 мг в день ежедневно, перорально, #тамоксифен** в дозе 10–20 мг в день ежедневно перорально, #фулвестрант** 4 мг/кг внутримышечно каждые 28 дней, #бикалутамид** в дозах от 12,5 до 100 мг/день по данным разных источников [140–148].

Диагноз «Преждевременное половое развитие» не значится в показаниях к применению ни у одного из вышеперечисленных препаратов, поэтому при назначении лекарственного препарата вне зарегистрированных показаний рекомендуется обязательное подробное информирование родителей с подписанием ими информированного согласия и инициация или согласование терапии в специализированных научных центрах с проведением врачебной комиссии [139].

Лечение гонадотропин-независимого преждевременного полового развития при синдроме МОБ у девочек назначается в случае опережения костного возраста на 2 года и более, частых эпизодов кровянистых выделений. В настоящее время рекомендуется назначение ингибитора ароматазы 3 поколения — #летрозол** 2,5 мг × 1 раз в день. При неэффективности данной терапии — сочетание или замена на антиэстрогены — #тамоксифен** 10 мг × 1 раз в день или #фулвестрант** 4 мг/кг/месяц [114][140–145].

Для лечения гонадотропин-независимого преждевременного полового развития при синдроме МОБ у мальчиков, сопровождающегося опережением костного возраста на 2 года и более, и для лечения тестотоксикоза в настоящее время рекомендуется назначение ингибитора ароматазы 3 поколения #анастрозол** 1 мг × 1 раз в день перорально сочетании с антиандрогенами #бикалутамидом** в дозах от 12,5 до 100 мг/день [146–148].

Возможна трансформация гонадотропин-независимого ППР в гонадотропин-зависимое при костном возрасте, близком к пубертатному. Для диагностики данного состояния используется стандартная проба с аналогом ГнРГ. При доказанном гонадотропин-зависимом характере ППР у детей с первично периферическим ППР возможно применение пролонгированных аналогов ГнРГ [11][149][150]. УДД — 3, УУР — B.

3.2. Хирургическое лечение.

3.3. Иное лечение.

Комментарии. Мальчики с ХГЧ-секретирующими герминативно-клеточными опухолями получают лечение у врачей-онкологов по соответствующим протоколам [156]. УДД — 3, УУР — B.

## 4. МЕДИЦИНСКАЯ РЕАБИЛИТАЦИЯ, МЕДИЦИНСКИЕ ПОКАЗАНИЯ И ПРОТИВОПОКАЗАНИЯ К ПРИМЕНЕНИЮ МЕТОДОВ РЕАБИЛИТАЦИИ

При отсутствии своевременного назначения лечения при преждевременном половом развитии отмечается ускоренное закрытие зон роста и негативный прогноз конечного роста, что может потребовать психологической поддержки для адаптации пациента.

## 5. ПРОФИЛАКТИКА И ДИСПАНСЕРНОЕ НАБЛЮДЕНИЕ, МЕДИЦИНСКИЕ ПОКАЗАНИЯ И ПРОТИВОПОКАЗАНИЯ К ПРИМЕНЕНИЮ МЕТОДОВ ПРОФИЛАКТИКИ

## 6. ОРГАНИЗАЦИЯ ОКАЗАНИЯ МЕДИЦИНСКОЙ ПОМОЩИ.

Показания для госпитализации в медицинскую организацию.

Показания к выписке пациента из медицинской организации:

## 7. ДОПОЛНИТЕЛЬНАЯ ИНФОРМАЦИЯ, ВЛИЯЮЩАЯ НА ТЕЧЕНИЕ И ИСХОД ЗАБОЛЕВАНИЯ.

На течение и исход заболевания влияют сроки манифестации заболевания, сроки установления диагноза, особенности основного заболевания, на фоне которого развилось преждевременного половое развитие

Б1. АЛГОРИТМ ДИАГНОСТИКИ ППР У ДЕВОЧЕК С ПРЕЖДЕВРЕМЕННЫМ УВЕЛИЧЕНИЕМ МОЛОЧНЫХ ЖЕЛЕЗ

**Table:** Таблица 3. Критерии оценки качества медицинской помощи

	Критерии качества	Уровень достоверности доказательств	Уровень убедительности рекомендаций
1.	Выполнены измерение роста и оценка SDS роста	УДД — 3, УУР — B.	УДД — 3, УУР — B
2.	Проведен подсчет скорости роста за предшествующие 6–12 мес (при наличии данных роста)	УДД — 3, УУР — B	УДД — 3, УУР — B
3.	Выполнена клиническая оценка полового развития на основании шкалы Таннер	УДД — 3, УУР — B	УДД — 3, УУР — B
4.	Выполнена рентгенография кистей с оценкой костного возраста при диагностике ППР	УДД — 3 УУР — C	УДД — 3, УУР — C
5.	Выполнено исследование уровня ЛГ крови при диагностике ППР	УДД — 3, УУР — B	УДД — 3, УУР — B
6.	У детей с преждевременным адренархе выполнено определение в крови 17ОНР, ДГЭА/ДГЭА-С при диагностике ППР	УДД — 3, УУР — B	УДД — 3, УУР — B
7.	Мальчикам с гонадотропин-зависимым ППР и девочкам с гонадотропин-зависимым ППР младше 6 лет выполнено МРТ головного мозга при диагностике ППР	УДД — 4, УУР — C	УДД — 4, УУР — C
8.	Выполнено УЗИ органов малого таза у девочек с ППР и УЗИ мошонки у мальчиков с ППР при диагностике ППР	УДД — 4, УУР — C	УДД — 4, УУР — C
9.	Проведены визуализирующие методы исследования надпочечников (УЗИ/МРТ/МСКТ) детям с адренархе и/или с повышенным уровнем ДГЭА и/или ДГЭА-С при диагностике ППР	УДД — 4, УУР — C	УДД — 4, УУР — C
10.	Девочкам младше 6 лет и мальчикам младше 9 лет назначен аналог Гн-РГ пролонгированного действия при доказанном центральном ППР	УДД — 5, УУР — C	УДД — 5, УУР — C
11.	Детям с центральным ППР, получающим лечение аналогом Гн-РГ пролонгированного действия, проводится регулярное (1 раз в 6–12 мес.) обследование для оценки эффективности терапии	УДД — 5, УУР — C	УДД — 5, УУР — C
12.	Детям с центральным ППР, получающим лечение аналогом Гн-РГ пролонгированного действия, при выявлении неэффективности проводимого лечения, проведена коррекция терапии.	УДД — 3, УУР — B	УДД — 3, УУР — B

**Figure:**
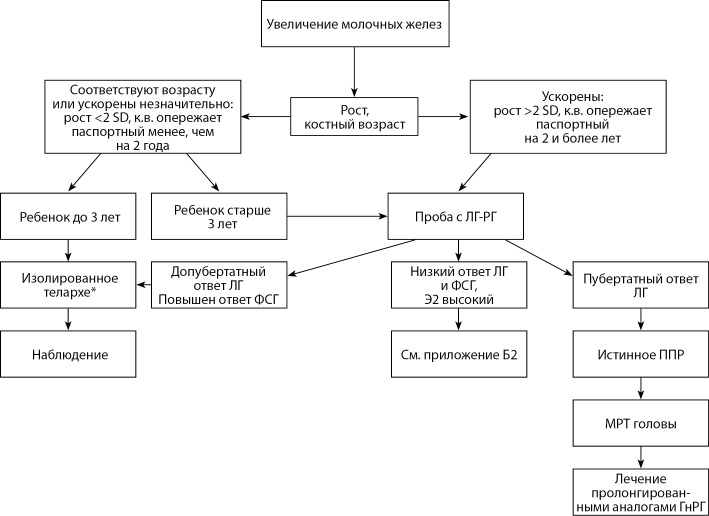


Б2. АЛГОРИТМ ДИАГНОСТИКИ ППР У ДЕВОЧЕК С ПРЕЖДЕВРЕМЕННЫМ УВЕЛИЧЕНИЕМ МОЛОЧНЫХ ЖЕЛЕЗ И МЕНСТРУАЛОПОДОБНЫМИ ВЫДЕЛЕНИЯМИ

**Figure:**
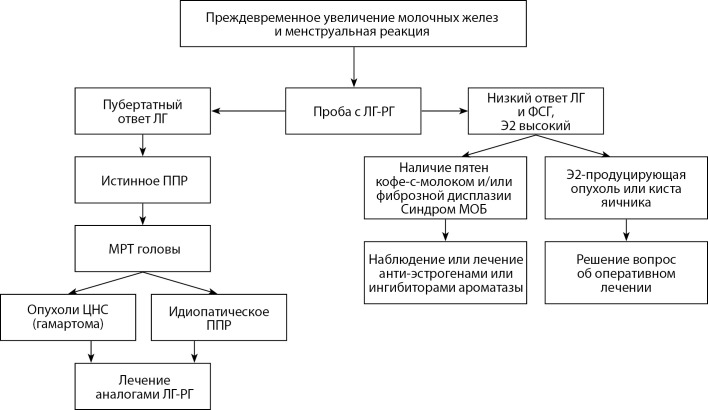


Б3. АЛГОРИТМ ДИАГНОСТИКИ ППР У ДЕВОЧЕК С ПРЕЖДЕВРЕМЕННЫМ ИЗОЛИРОВАННЫМ АДРЕНАРХЕ

**Figure:**
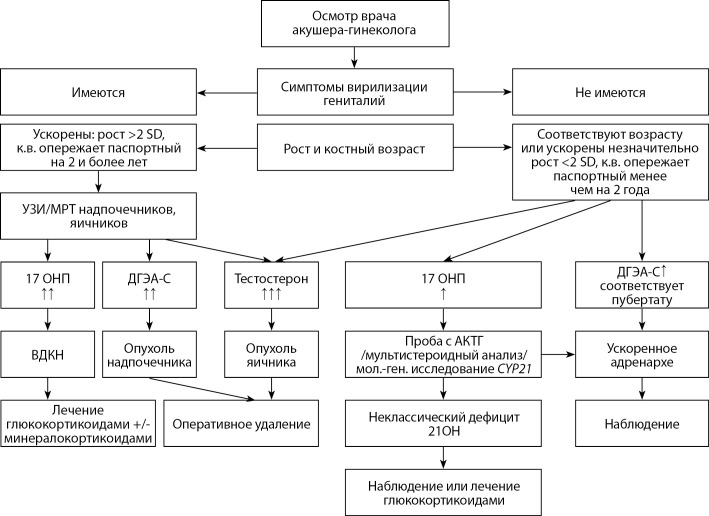


Б4. АЛГОРИТМ ДИАГНОСТИКИ ППР У МАЛЬЧИКОВ

**Figure:**
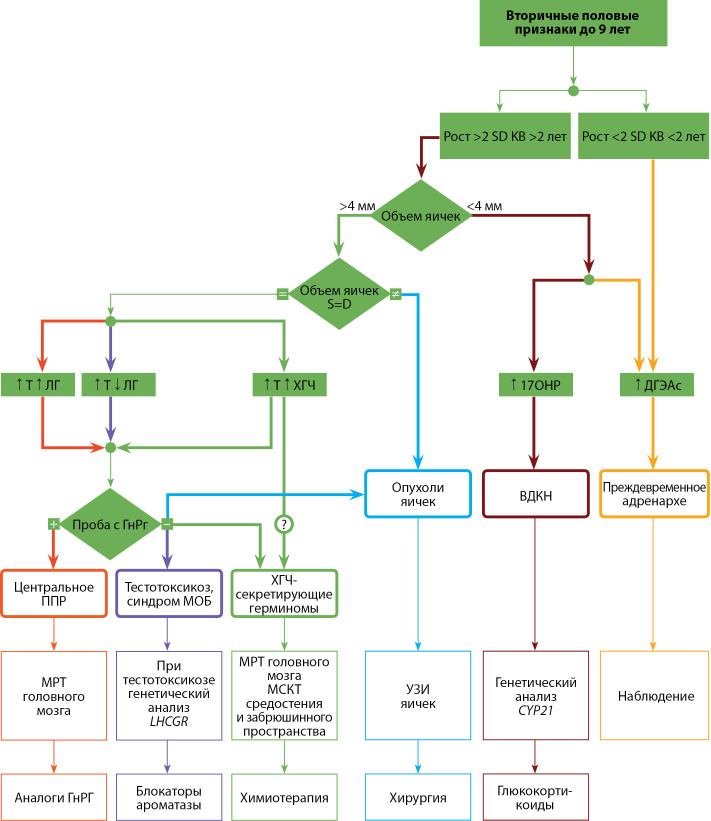


## ДОПОЛНИТЕЛЬНАЯ ИНФОРМАЦИЯ

Порядок обновления клинических рекомендаций. Механизм обновления клинических рекомендаций предусматривает их систематическую актуализацию — не реже чем 1 раз в 3 года, а также при появлении новых данных с позиции доказательной медицины по вопросам диагностики, лечения, профилактики и реабилитации конкретных заболеваний, наличии обоснованных дополнений/замечаний к ранее утвержденным КР, но не чаще 1 раза в 6 мес.

Источники финансирования. Работа выполнена по инициативе авторов без привлечения финансирования.

Конфликт интересов. Авторы декларируют отсутствие явных и потенциальных конфликтов интересов, связанных с содержанием и публикацией настоящей статьи.

Участие авторов. Все авторы одобрили финальную версию статьи перед публикацией, выразили согласие нести ответственность за все аспекты работы, подразумевающую надлежащее изучение и решение вопросов, связанных с точностью или добросовестностью любой части работы.
